# Evaluation of the chitin-binding dye Congo red as a selection agent for the isolation, classification, and enumeration of ascomycete yeasts

**DOI:** 10.1007/s00203-018-1498-y

**Published:** 2018-02-23

**Authors:** Tomas Linder

**Affiliations:** Department of Molecular Sciences, Swedish University of Agricultural Sciences, Box 7015, 750 07 Uppsala, Sweden

**Keywords:** Antifungal, Cell wall, Phenotype, Yeast

## Abstract

Thirty-nine strains of ascomycete yeasts representing 35 species and 33 genera were tested for their ability to grow on solid agar medium containing increasing concentrations of the chitin-binding dye Congo red. Six strains were classified as hypersensitive (weak or no growth at 10 mg/l Congo red), five were moderately sensitive (weak or no growth at 50 mg/l), three were moderately tolerant (weak or no growth at 100 mg/l), while the remaining 25 strains were classified as resistant (robust growth at ≥ 100 mg/l) with 20 of these strains classified as hyper-resistant (robust growth at 200 mg/l). Congo red growth phenotypes were consistent within some families but not others. The frequency of Congo red resistance among ascomycete yeasts was deemed too high for the practical use of Congo red as a selection agent for targeted isolation, but can be useful for identification and enumeration of yeasts.

## Introduction

The development of genome sequencing over the past four decades has revolutionized yeast taxonomy and now enables nearly unambiguous species identification (Hittinger et al. 2015). More recent developments in spectroscopic methods also allows for rapid and direct identification of yeast isolates (Quintilla et al. 2017). Before the advent of these technologies, yeast species were exclusively identified using morphological and physiological characterization (Kurtzman et al. 2011). However, these methods are still widely used in many parts of the world, where sequencing or spectroscopic facilities are not readily available.

Physiological properties of yeasts can also be used for targeted isolation of particular taxonomic groups. Chemically defined cultivation media that contain either selective growth inhibitors or selected carbon and/or nitrogen sources can be used for targeted isolation of certain taxonomic groups of yeasts. For example, chemically defined growth medium containing methanol as the sole carbon source is commonly used to isolate species of methylotrophic yeasts (van Dijken and Harder 1974). A third use of physiological properties of yeasts is the enumeration of viable yeast cells in mixed cultures using colony counting assays, which requires the use of selective media to distinguish individual yeast strains and species from each other.

The anionic azo dye Congo red (disodium benzidinediazo-bis-1-napthylamine-4-sulfonate) was originally developed for the textile industry (Steensma 2001) and is used routinely to stain chitin in microscopy preparations of fungi. Exposure of living cells of the baker’s yeast *Saccharomyces cerevisiae* to Congo red causes retarded growth and cell separation defects (Roncero and Durán 1985; Kopecká and Gabriel 1992). The exact mechanism of growth inhibition is unclear, but one hypothesis is that the binding of Congo red to chitin impairs cell wall rigidity by interfering with the formation of covalent links between chitin chains and β-glucan (Ram and Klis 2006).

A survey of the published literature failed to return any previous studies on relative Congo red tolerance among different taxonomic groups of ascomycete yeast. The current study, therefore, set out to investigate the diversity of Congo red tolerance among ascomycete yeasts to determine whether it could serve as a useful selection agent for yeast isolation, classification, and/or enumeration.

## Materials and methods

### Yeast strains, cultivation media, and reagents

**Fig. 1:**
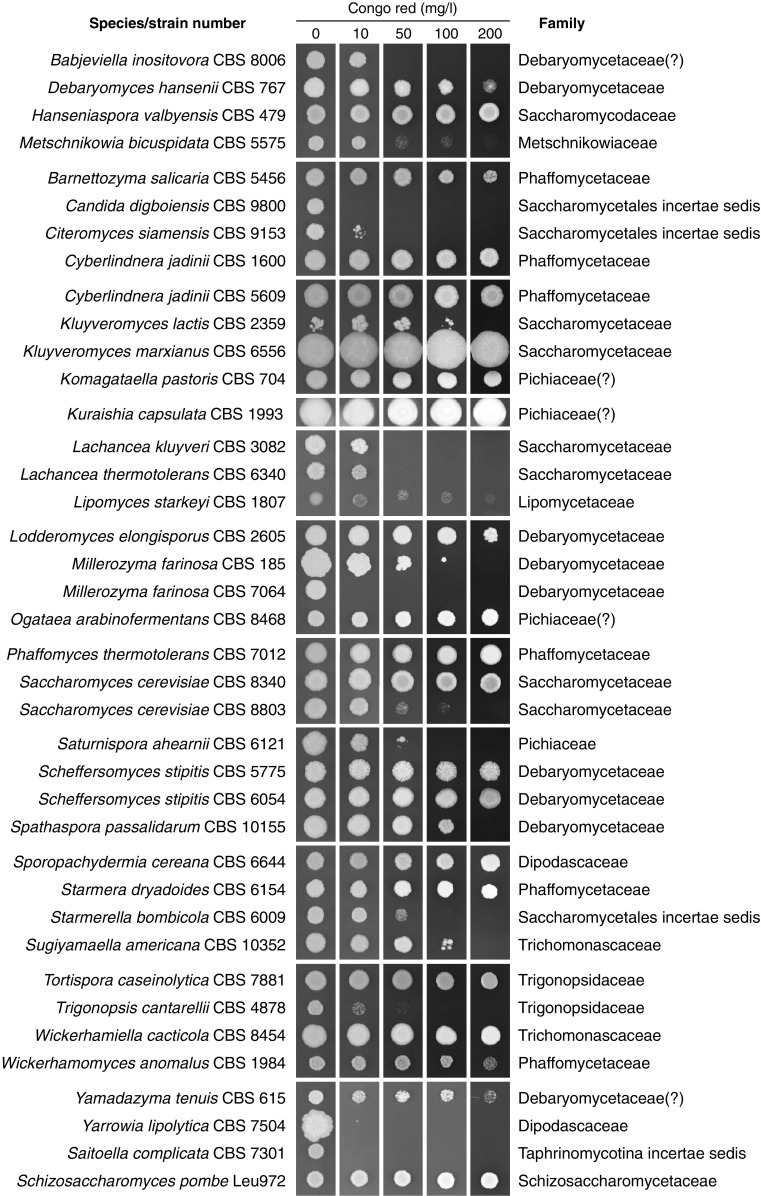
Congo red agar plate growth assay. Family assignments were based on the NCBI Taxonomy browser (https://www.ncbi.nlm.nih.gov/taxonomy) and a recent genome-scale phylogenetic analysis of budding yeasts (Shen et al. 2016)

The yeast strains used in this study are listed in Fig. 1. *Schizosaccharomyces pombe* strain Leu972 was a kind gift from Dr. Pernilla Bjerling (Uppsala University, Sweden). All other strains were purchased from Centraalbureau voor Schimmelcultures (Utrecht, The Netherlands). Strains were maintained on YM agar (3 g/l yeast extract, 3 g/l malt extract, 5 g/l peptone, 10 g/l glucose, and 20 g/l agar) supplemented with 15 mg/l chloramphenicol to prevent bacterial contamination. Yeast plates were stored at 4 °C with strains re-streaked on fresh YM agar approximately every 4 weeks. A stock solution of Congo red (Merck) was prepared in water to a final concentration of 10 g/l and sterilized by filtration. Congo red stock solutions were stored short-term at room temperature in the dark. YM agar plates containing Congo red were stored at 4 °C in the dark and were typically used within 1 week of preparation.

### Congo red tolerance assays

Yeast strains were pre-cultured overnight in 3 ml YM broth (3 g/l yeast extract, 3 g/l malt extract, 5 g/l peptone, and 10 g/l glucose) supplemented with 15 mg/l chloramphenicol. The following day each pre-culture was diluted to optical density 0.1 at 600 nm in fresh YM broth. 2 µl diluted cell suspension of each strain was spotted on YM agar with the indicated concentration of Congo red. Plates were incubated for 3 days in the dark and then photographed. YM agar plates containing *Babjeviella inositovora, Debaryomyces hansenii, Hanseniaspora valbyensis*, and *Metschnikowia bicuspidata* were incubated at 25 °C. All other strains were incubated at 30 °C.

## Results and discussion

To the best of the author’s knowledge, no extensive study of sensitivity or tolerance to Congo red among ascomycete yeasts has been carried out to date. The lack of published data on Congo red growth phenotypes among yeasts as well as a rudimentary understanding of possible genetic factors underlying these phenotypes made prediction difficult. The fission yeast *S. pombe* has previously been reported to be resistant to the chitin-binding dye Calcofluor white (Roncero and Durán 1985), which is likely due to the absence of chitin in the cell walls of this yeast (van der Klei et al. 2011). It was, therefore, expected that *S. pombe* would display resistance to Congo red as well.

The usefulness of Congo red as a selection agent for the purposes of isolation, classification, and enumeration would depend on the frequency of Congo red resistance (defined here as moderate-to-strong growth at Congo red concentration at or above 100 mg/l) among yeasts. Three possible scenarios were considered. A scenario where Congo red resistance would be rare (< 20% of strains tested) would be suitable for targeted isolation and enumeration of the resistant lineages but less useful for classification purposes. A second scenario with an intermediate frequency of Congo red resistance (20–80% of strains tested) would be useful for classification and enumeration but would have to be combined with other selective determinants (e.g., carbon or nitrogen source) for targeted isolations. A third scenario where resistance to Congo red is found in the majority (> 80%) of tested strains would limit the usefulness of this trait for the purposes of isolation, classification, and enumeration altogether.

As the physiological determinants of Congo red growth phenotypes in yeast are poorly understood, another goal of the current study was to investigate whether growth phenotypes would be consistent within higher taxonomic ranks such as genera or families. If Congo red growth phenotypes are predominantly determined by fundamental properties of the cell wall (such as the lack of chitin in the cell wall of the genus *Schizosaccharomyces*), growth phenotypes are expected to be consistent throughout taxonomic ranks such as genera or families. Conversely, if Congo red growth phenotypes are predominantly determined by randomly occurring alleles, greater variation would be expected within taxonomic ranks.

Thirty-nine strains representing 33 species of budding yeasts (sub-phylum Saccharomycotina) and two species of fission yeasts (sub-phylum Taphrinomycotina) were selected for the study. In the case of budding yeasts, an effort was made to select representative species spanning the entire sub-phylum (Hittinger et al. 2015). Four species of budding yeasts (*Cyberlindnera jadinii, Millerozyma farinosa, S. cerevisiae*, and *Scheffersomyces stipitis*) were represented by two strains each to investigate whether intra-species variation in Congo red growth phenotype occurs. Similarly the genera *Kluyveromyces* and *Lachancea* were represented by two species each to investigate intra-genus variation in Congo red growth phenotypes.

Following a 3-day incubation on agar medium with different concentrations of Congo red, the strains were classified as either hypersensitive (weak or no growth at 10 mg/l Congo red), moderately sensitive (weak or no growth at 50 mg/l), moderately tolerant (weak or no growth at 100 mg/l), resistant (weak or no growth at 200 mg/l), or hyper-resistant (robust growth at 200 mg/l). Six strains were classified as hypersensitive, five strains were moderately sensitive, three were moderately tolerant, five strains were resistant, and 20 strains were hyper-resistant (Fig. 1). As predicted, *S. pombe* displayed hyper-resistance to Congo red.

The four pairs of species represented by duplicate strains displayed notably different Congo red growth phenotypes. In the case of *C. jadinii* and *S. stipitis*, Congo red hyper-resistance was observed in both pairs of strains while the strains of *M. farinosa* and *S. cerevisiae* displayed significant intra-species difference in Congo red sensitivity. *M. farinosa* strain CBS 185 displayed Congo red resistance while strain CBS 7064 appeared hypersensitive. *M. farinosa* CBS 7064 is a recently formed interspecies hybrid (Louis et al. 2012) that has been shown to be phylogetically distinct from the CBS 185 strain within the *M. farinosa* species complex (Mallet et al. 2012). Future characterization of additional *M. farinosa* strains in combination with genome sequencing may shed light on the underlying physiological properties that determine Congo red sensitivity in this species. In the case of *S. cerevisiae*, strains CBS 8803 (syn. S288C) and CBS 8340 (syn. CEN.PK113-7D) are both considered domesticated and may, therefore, not reflect the Congo red growth phenotypes that would be found in wild isolates. Nevertheless, the availability of genomic sequences for both strains would enable studies of genetic determinants underlying the difference in Congo red growth phenotypes between these strains. The genomes of strains CBS 8803 and CBS 8340 have been shown to differ by 21,889 single-nucleotide variations of which 13,235 were mapped to coding regions (Nijkamp et al. 2012).

The two genera represented by more than one species displayed fairly consistent Congo red growth phenotypes. Both species of *Lachancea* displayed equal sensitivity to Congo red (no detectable growth at ≥ 50 mg/l). Of the two species of *Kluyveromyces* included in the present study, *Kluyveromyces marxianus* displayed hyper-resistance to Congo red while *Kluyveromyces lactis* was unable to grow at 200 mg/l. In cases where there was more than a one species representing a family, Congo red growth phenotypes were fairly consistent in some cases (Debaryomycetaceae, Phaffomycetaceae, Pichiaceae, and Trichomonascaceae) while displaying great variation in others (Dipodascaceae, Saccharomycetaceae, and Trigonopsidaceae).

It is clear that the variability of Congo red growth phenotypes within taxonomic ranks such as families, genera, and species needs to be investigated further. The notable variation in Congo red growth phenotypes among ascomycete yeasts may reflect fundamental cell wall properties within different taxonomic groups, many of which have yet to be studied in depth. Another question is what the role individual alleles play in determining Congo red tolerance and sensitivity in ascomycete yeasts. Sensitivity to Congo red has previously been associated with an impaired cell wall stress response in yeast (García et al. 2004; Ram and Klis 2006). However, several of the Congo red sensitive strains used in this study are wild isolates and as such are expected to have been under active selection in environments shared with microorganisms that produce antagonistic secondary metabolites targeting fungal cell wall synthesis and function. It would, therefore, be informative to study whether Congo red growth phenotypes correlate with those of cell wall synthesis inhibitors such as nikkomycin or echinocandins.

In conclusion, the present study has demonstrated a clear diversity in Congo red growth phenotypes among ascomycete yeasts. Although Congo red tolerance (growth at concentrations ≥ 100 mg/l) is too common to be useful for targeted isolation, there appears to be sufficient variation among taxonomic groups to motivate the use of Congo red for the purposes of classification and enumeration of ascomycete yeasts.

## References

[CR1] García R, Bermejo C, Grau C, Pérez R, Rodríguez-Peña JM, Francois J, Nombela C, Arroyo J (2004). The global transcriptional response to transient cell wall damage in *Saccharomyces cerevisiae* and its regulation by the cell integrity signaling pathway. J Biol Chem.

[CR2] Hittinger CT, Rokas A, Bai FY, Boekhout T, Gonçalves P, Jeffries TW, Kominek J, Lachance MA, Libkind D, Rosa CA, Sampaio JP, Kurtzman CP (2015). Genomics and the making of yeast biodiversity. Curr Opin Genet Dev.

[CR3] Kopecká M, Gabriel M (1992). The influence of Congo red on the cell wall and (1→3)-β-d-glucan microfibril biogenesis in *Saccharomyces cerevisiae*. Arch Microbiol.

[CR4] Kurtzman CP, Fell JW, Boekhout T, Robert V, Kurtzman CP, Fell JW, Boekhout T (2011). Methods for isolation, phenotypic characterization and maintenance of yeasts. The yeasts, a taxonomic study.

[CR5] Louis VL, Despons L, Friedrich A, Martin T, Durrens P, Casarégola S, Neuvéglise C, Fairhead C, Marck C, Cruz JA, Straub ML, Kugler V, Sacerdot C, Uzunov Z, Thierry A, Weiss S, Bleykasten C, De Montigny J, Jacques N, Jung P, Lemaire M, Mallet S, Morel G, Richard GF, Sarkar A, Savel G, Schacherer J, Seret ML, Talla E, Samson G, Jubin C, Poulain J, Vacherie B, Barbe V, Pelletier E, Sherman DJ, Westhof E, Weissenbach J, Baret PV, Wincker P, Gaillardin C, Dujon B, Souciet JL (2012). *Pichia sorbitophila*, an interspecies yeast hybrid, reveals early steps of genome resolution after polyploidization. G3.

[CR6] Mallet S, Weiss S, Jacques N, Leh-Louis V, Sacerdot C, Casaregola S (2012). Insights into the life cycle of yeasts from the CTG clade revealed by the analysis of the Millerozyma (Pichia) farinosa species complex. PLoS One.

[CR7] Nijkamp JF, van den Broek M, Datema E, de Kok S, Bosman L, Luttik MA, Daran-Lapujade P, Vongsangnak W, Nielsen J, Heijne WH, Klaassen P, Paddon CJ, Platt D, Kötter P, van Ham RC, Reinders MJ, Pronk JT, de Ridder D, Daran JM (2012). De novo sequencing, assembly and analysis of the genome of the laboratory strain *Saccharomyces cerevisiae* CEN.PK113-7D, a model for modern industrial biotechnology. Microb Cell Fact.

[CR8] Quintilla R, Kolecka A, Casaregola S, Daniel HM, Houbraken J, Kostrzewa M, Boekhout T, Groenewald M (2017). MALDI-TOF MS as a tool to identify foodborne yeasts and yeast-like fungi. Int J Food Microbiol.

[CR9] Ram AF, Klis FM (2006). Identification of fungal cell wall mutants using susceptibility assays based on Calcofluor white and Congo red. Nat Protoc.

[CR10] Roncero C, Durán A (1985). Effect of Calcofluor white and Congo red on fungal cell wall morphogenesis: in vivo activation of chitin polymerization. J Bacteriol.

[CR11] Shen XX, Zhou X, Kominek J, Kurtzman CP, Hittinger CT, Rokas A (2016). Reconstructing the backbone of the Saccharomycotina yeast phylogeny using genome-scale data. G3.

[CR12] Steensma DP (2001). “Congo” red: out of Africa?. Arch Pathol Lab Med.

[CR13] van Dijken JP, Harder W (1974). Optimal conditions for the enrichment and isolation of methanol-assimilating yeasts. J Gen Microbiol.

[CR14] van der Klei I, Veenhuis M, Brul S, Klis FM, De Groot PWJ, Müller WH, Driel KGA, Boekhout T, Kurtzman CP, Fell JW, Boekhout T (2011). Cytology, cell walls and septa: a summary of yeast cell Biology from a phylogenetic perspective. The yeasts, a taxonomic study.

